# Trabeculectomy with Healaflow versus Trabeculectomy for the Treatment of Glaucoma: A Case-Control Study

**DOI:** 10.1155/2015/836269

**Published:** 2015-06-11

**Authors:** Dimitris Papaconstantinou, Andreas Diagourtas, Petros Petrou, Alexandros Rouvas, Athanasios Vergados, Chryssanthi Koutsandrea, Ilias Georgalas

**Affiliations:** Department of Ophthalmology, “G. Gennimatas” Hospital, University of Athens, Mesogeion Avenue, 11527 Athens, Greece

## Abstract

*Purpose*. To compare the outcomes of trabeculectomy with and without Healaflow (Anteis S.A, Geneva, Switzerland), a high molecular weight viscoelastic gel, in patients requiring glaucoma surgery. *Methods*. This was a retrospective, comparative, interventional case-control study. Forty patients formed two matched study groups and were analyzed (trabeculectomy alone (control) versus trabeculectomy with Healaflow (study)). *Results*. The postoperative levels of mean IOP were statistically significantly lower (*P* < 0.05) than preoperatively in both groups, for all time intervals. There was no statistical difference, at the end of the follow-up period, between the two groups in the mean values of the IOP (14.9 ± 3.2 mmHg for the study group versus 14.8 ± 3.3 mmHg for the control group). The number of antiglaucoma drugs used in the study group was reduced from a preoperative mean of 3.4 ± 0.75 to a 6-month postoperative mean of 0.6 ± 0.8 (*P* < 0.001) and in the control group from 3.6 ± 0.59 to 0.55 ± 0.9 (*P* < 0.001). *Conclusions*. Although trabeculectomy with Healaflow appears to be a safe procedure, we failed to identify any significant advantages in the use of Healaflow when compared with trabeculectomy alone, at the end of the 6-month follow-up period.

## 1. Introduction

Since its introduction in 1968 trabeculectomy is the most common incisional surgery for the treatment of glaucoma [1–4]. However, scar tissue deposition at the level of the conjunctival-Tenon-episcleral interface, due to the healing process, may result in fibrosis of the formatted bleb and obstruction of the fistula, leading eventually to operation's failure and poor postoperative IOP control [5, 6]. It is well documented that inhibition of scar formation during the fibrotic cascade should promote greater success [5, 7].

Pathology studies have shown that, following trabeculectomy, migration, proliferation, and differentiation of the fibroblasts at the subconjunctival level, which are responsible for the fibrotic process and eventually the bleb failure, occur as early as the third to fifth postoperative day [8, 9]. Adjunctive antimetabolites, such as 5-fluorouracil (5-FU) and mitomycin C (MMC), both intra- and postoperatively, are commonly used to enhance the long-term success of trabeculectomy [10–14]. However, due to their nonselective cytotoxicity, which could lead to conjunctival barrier breakdown, their use is not free of increased risk of complications, some of them devastating, such as ischemic and leaking bleb which could predispose to serious infections and even endophthalmitis [15–19].

Multiple other agents such as corticosteroids, growth factor inhibitors (anti-TGF*β*2), amniotic membranes, collagen-glycosaminoglycan matrix implants, and, lately, vascular endothelial growth factor inhibitors (anti-VEGF) and matrix metalloproteinase inhibitors (anti-MMPs) have been applied intraoperatively or postoperatively as wound healing modulators, at the site of the sclera flap, intracamerally or subconjunctivally, in order to enhance the long-term outcomes of filtration surgery and minimize the adverse effects of the antimetabolites, usually with controversial results [20–29].

This study aimed to evaluate the effectiveness and safety profile of a new cross-linked viscoelastic agent, Healaflow (Anteis S.A, Geneva, Switzerland), used as adjunctive material intraoperatively. This high molecular weight, sodium hyaluronate material, injected in the conjunctival-Tenon-episcleral interface, is considered to offer space-occupying effect during the early postoperative period, on the one hand preventing the accumulation of fibroblasts and the deposition of collagen and on the other hand minimizing the risk of overfiltration and hypotony-hypothalamia.

With its anti-inflammatory properties this slowly resorbable material is considered to reduce the healing process cascade [30], responsible for bleb encapsulation and trabeculectomy failure.

The primary outcome of the study was to compare the hypotensive results of trabeculectomy with adjunctive Healaflow versus trabeculectomy alone, in patients requiring glaucoma surgery for uncontrolled IOP and/or visual field progression, and the secondary one was comparing the number of postoperative antiglaucoma medications used and the complications rate between the two groups.

## 2. Methods

The present work was a retrospective, comparative, interventional case-control study and was conducted during the period between November 2012 and April 2013 at the Ophthalmology Department of the University of Athens, Greece (“G. Gennimatas” Hospital). Approval was obtained from the local Committee on Research Ethics, and informed consent according to the tenets of the Declaration of Helsinki was obtained from all subjects.

**Figure 1:**
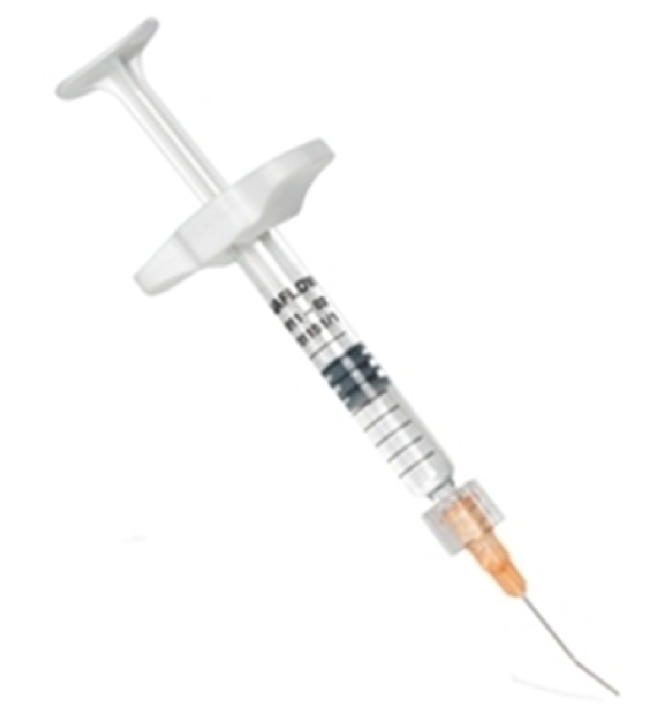
The Healaflow injectable implant.

Patients were included in the analysis provided that they met the inclusion criteria (age > 18 years, glaucoma requiring trabeculectomy with or without Healaflow (Figure 1) for IOP control, and at least six-month postoperative follow-up). Data from only the selected eye of each eligible patient was used for the study. Patients were excluded from the analysis if there was a history of neovascular, uveitic, and posttraumatic glaucoma or a history of previous surgical interventions or laser procedures for glaucoma. In addition, patients with retinal or neurological comorbidities that may have affected the visual function were also excluded from the analysis.

Twenty consecutive eligible patients (trabeculectomy with Healaflow (study group)) were matched for age, gender, visual acuity, eye laterality, type of glaucoma, preoperative IOP, and number of prescribed topical or systemic antiglaucoma medications. The forty patients formed the two study groups and were analyzed (trabeculectomy alone (control) versus trabeculectomy with Healaflow (study)).

Table 1 summarizes the technical sheet of Healaflow. The study was approved by the institutional review board of the hospital.

For each patient, pre-, intra-, and postoperative data were collected.

*Preoperative Characteristics.* Preoperative characteristics were age, gender, type of glaucoma, IOP, and number of preoperative glaucoma medications used during a period of three months before surgery.

*Intraoperative Characteristics.* Intraoperative characteristics were date of operation, surgical technique, and presence of any intraoperative complications.

*Postoperative Characteristics.* IOP, complications, and number of postoperative glaucoma medications were recorded. All IOP measurements were performed using the Goldmann applanation tonometer.

The primary study outcome was surgical success defined as complete (1) if the IOP measured 18 mmHg or less without antiglaucoma medications and as qualified (2) if the IOP measured 18 mmHg or less with or without antiglaucoma medications provided that no further surgical procedures (reinterventions) had to be performed. Secondary study outcomes included the number of postoperative glaucoma medications, postoperative complications, and number of needling procedures required in each group.

Hypotony was defined as an IOP less than 6 mmHg. Flat anterior chamber was defined as iridocorneal touch with a depth of less than one unit equal to corneal thickness centrally.

### 2.1. Surgical Technique

All surgical procedures were performed by the same surgeon (D.P.). After peribulbar anesthesia with lidocaine 1%, the eye was prepared and draped. A 7-0 silk traction suture was placed in the upper cornea adjacent to limbus and a fornix-based conjunctival flap centered at 12 o'clock was prepared. A one-half thickness rectangular scleral flap of approximately 3.5 × 3.5 mm was dissected extending 1 mm from the limbus, followed by an anterior chamber paracentesis. A sclerostomy of 1 × 2 mm with a knife was followed by a peripheral iridectomy. The scleral flap was sutured with two 10-0 nylon sutures, one in each corner.

In the study group, just before the conjunctival closure, a small quantity (0.05–0.1 mL) of Healaflow was injected under the scleral flap with care not to pass intracamerally, in order to avoid pressure spikes, and a larger amount (0.2–0.4 mL) was injected between the sclera and the conjunctiva, which was sutured with two 10-0 nylon sutures anchored at the limbus. No intraoperative mitomycin C or 5-FU was used in either group. Postoperatively a topical fixed combination of chloramphenicol and dexamethasone 1 mg/mL was administered four times daily for four weeks and then gradually tapered over 6 to 8 weeks. Also atropine 1% was used three times daily for two weeks in both groups.

### 2.2. Statistical Analysis

Statistical analysis was performed with SPSS (SPSS Inc., Chicago, IL). Demographic and preoperative data were analyzed with Student's *t*-test or *χ*² test. Intraocular pressure comparisons between the 2 groups were analyzed with paired *t*-test. Rates of surgical success, surgical failure, and postoperative complications were analyzed by Fisher's exact test or the *χ*² test, when applicable. Kaplan-Meier survival analysis for success (complete or qualified) was calculated with the log-rank test. Statistical significant level was set at *P* < 0.05. In cases where additional procedures had to be performed, the IOP values measured at the prescheduled follow-up visits were used for the statistical analysis.

## 3. Results

Forty eyes of 40 consecutive eligible patients were enrolled in the study. All patients completed the 6-month postoperative follow-up visit (as per inclusion criteria) and the mean follow-up period was 8.5 months (range 6–18 months).

### 3.1. Preoperative Characteristics

Table 2 summarizes the main demographic and preoperative data of the two groups. Overall, with regard to the type of glaucoma, 19 eyes were identified with primary open angle glaucoma (POAG), 11 with pseudoexfoliative glaucoma (PXG), 2 with pigmentary glaucoma (PG), 4 with pseudophakic glaucoma, and 4 with primary angle closure glaucoma (PACG). The two groups were matched for age, gender, visual acuity, eye laterality, type of glaucoma, preoperative IOP, and number of prescribed topical or systemic antiglaucoma medications.

### 3.2. Intraoperative Characteristics

No intraoperative complications regarding the trabeculectomy and the injectable implant were detected.

### 3.3. Postoperative Characteristics

Mean IOP for both groups during follow-up visits is listed in Figure 4. No differences were observed in IOP during the follow-up period. Postoperative IOP levels were significantly lower than the respective preoperative levels for both groups at all intervals (*P* < 0.05). The IOP was lower than 18 mmHg at the six-month follow-up visit both for the control and for the study eyes.

### 3.4. Primary Study Outcome

**Figure 2:**
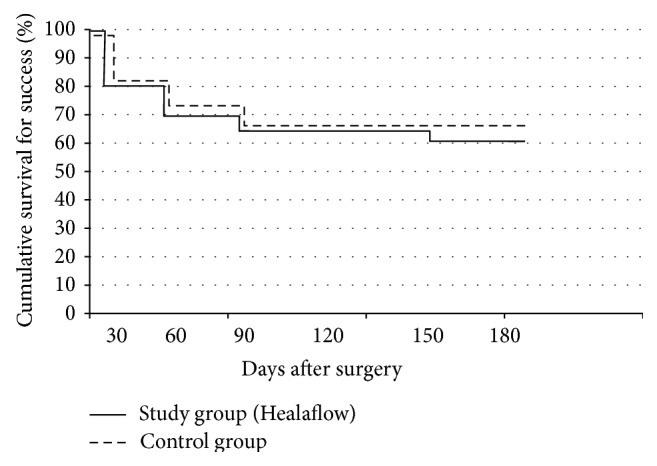
Kaplan-Meier survival analysis for both groups using the complete success definition (IOP ≤ 18 mmHg without antiglaucoma medications).

Figure 2 presents Kaplan-Meier survival analysis for both groups using the complete success definition. No statistically significant difference was observed between the survival curves in follow-up period. Complete success at six months after surgery was observed in 12 (60%) study eyes and in 13 (65%) control eyes. Qualified success at six months after surgery was observed in all (100%) study eyes and in all (100%) eyes in the control group.

### 3.5. Secondary Study Outcomes

The number of antiglaucoma medications used in the study group was reduced from a preoperative mean of 3.4 ± 0.75 to a 6-month postoperative mean of 0.6 ± 0.8 (*P* < 0.001) and in the control group from 3.6 ± 0.59 to 0.55 ± 0.9 (*P* < 0.001). The number of antiglaucoma drugs used at any interval of the follow-up period, in the two groups, did not differ significantly.

Table 3 summarizes the postoperative complications recorded in both groups. No statistically significant differences between groups were observed.

One eye with positive Seidel test and flat anterior chamber in the study group was managed with the placement of a bandage contact lens, covering the conjunctival area of leak. After four days the chamber was reformed and the leak was resolved. Flat anterior chamber in the control group was transient and settled spontaneously.

The occurrence of an encapsulated bleb was observed in four study eyes and five control eyes by the end of the first postoperative month. This was managed with 5-fluorouracil (5-FU) as adjuvant therapy, in a dose of 5 mg, injected twice weekly, via a 30-gauge needle, in the area adjacent to the peripheral limit of the bleb. The frequency of the injections was determined in each case by the main investigator. The number of injections varied from 1 to 8 (Table 4). No statistical significance was detected between the total doses of 5-FU between the two groups (*P* = 0.275). No laser suture lysis was needed in both groups.

**Figure 3:**
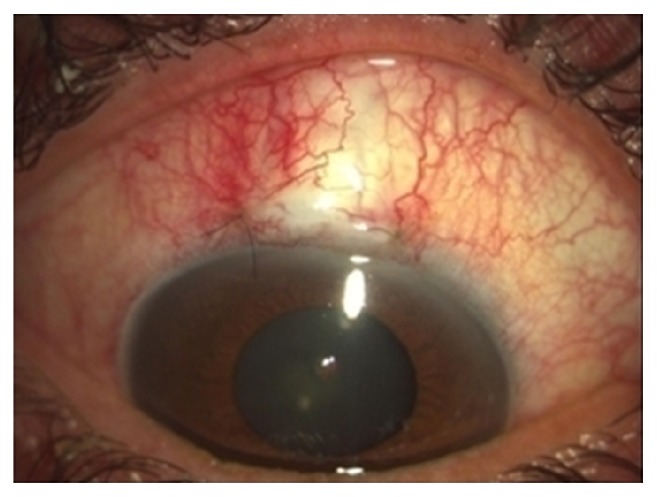
Three weeks after trabeculectomy with Healaflow (the injectable material is mostly absorbed).

**Figure 4:**
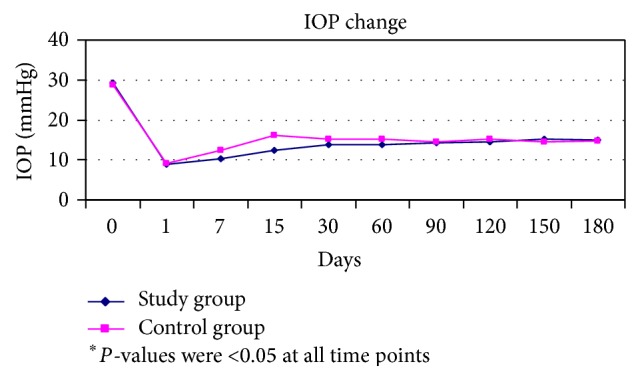
Graph representing the mean pre- and postoperative IOP for the study (patients who underwent trabeculectomy with Healaflow) and the control (patients who underwent trabeculectomy) groups (mmHg).

Figure 3 demonstrates the clinical appearance three weeks after trabeculectomy with Healaflow.

## 4. Discussion

Trabeculectomy is the most common surgical procedure for the treatment of glaucoma until today, although the long-term results are less than optimal and the rate of complications is relatively high, both in the early and in the late postoperative period. This filtering procedure is aiming to remove a portion of the trabecular meshwork, creating a fistula between the anterior chamber and the subconjunctival space. The necessary sclerostomy is covered by a partial-thickness sclera flap [2, 31].

During the early postoperative period, excessive aqueous filtration could lead to low intraocular pressure and eventually cause a number of complications. The most frequent undesirable event observed is the choroidal detachment, which usually resolves spontaneously. Hypotony, especially if occurring in phakic patients and followed by flat anterior chamber, could lead to corneal decompensation and cataract. If it persists for more than 3 months it can be associated with maculopathy and loss of visual acuity [32–34].

On the contrary, wound healing process and scar formation, mediated mainly by the fibroblasts and a cascade of biological events, at the area surrounding the fistula, could result in bleb encapsulation and obstruction of aqueous outflow, leading to failure of the trabeculectomy [35–37]. The long-term survival has been improved with the use of intraoperative or postoperative antimetabolites as adjuvant [38–40]. However, such antimitotic drugs interfere indiscriminately with cellular proliferation. The use of mitomycin C (MMC) or 5-fluorouracil (5-FU) has been associated with slow damage of the conjunctiva, leading sometimes to leakage and secondary infection, even many years after the operation [41–43]. In an attempt for more targeted approach and in order to avoid such complications multiple other agents and materials, such as corticosteroids, growth factor inhibitors, matrix metalloproteinase inhibitors, amniotic membrane, and collagen implants, have been applied to enhance the results of trabeculectomy [20–27]. Anti-VEGF agents are also becoming popular during filtering procedures, as novel wound modulating material, due to their anti-inflammatory and antiangiogenic effects [28, 29]. However most of the published studies are of small sample size, the follow-up period usually is short, and the results are controversial.

Recently, it has been reported in the literature [44] that the use of a slowly resorbable cross-linked viscoelastic gel, both in trabeculectomies and in nonpenetrating filtering surgeries, injected under the scleral flap and between the sclera and the conjunctiva, offers a long-lasting hypotensive effect as it stabilizes a patent scleral lake and minimizes filtering bleb encapsulation by preventing adhesions between flap and sclera and between conjunctiva and episcleral tissue. The Healaflow, according to the manufacturing company, has additionally an anti-inflammatory effect [30], because it inhibits cytokines, cell migration, phagocytosis, and lymphocyte transformation. In addition, it is nontoxic and highly purified and has less risk of allergy caused by animal proteins. The injectable implant is also sterile, colorless, and totally transparent. The application of Healaflow in our study was performed according to the manufacturing company instructions.

In our study, bleb vascularity was similar in both groups during the follow-up period and the only noticeable morphologic difference was the more prominent appearance of bleb in the study group during the first three operative weeks. We postulate that, following that period, sodium hyaluronate agent was gradually absorbed.

All eyes (in both control and study group) demonstrated significant reduction in the IOP throughout the postoperative period at all intervals (Table 2). The number of antiglaucoma medications employed in both groups was reduced after surgery significantly. However, regarding the mean postoperative IOP or the number of glaucoma medications, no statistically significant difference was found between the study groups.

In addition, there was no statistically significant difference between the 2 groups regarding the number and type of postoperative complications.

The occurrence of encapsulated bleb was encountered more frequently (5 eyes) in the control group when compared to the study group (4 eyes); however this difference was not statistically significant. In all cases 5-FU as adjuvant therapy was administered and none of the patients required further surgery.

With regard to the presence of postoperative hypotony, we found no significant difference between the two groups. Clinical examination revealed a positive Seidel test in one study eye and additional placement of a bandage contact lens was needed, in order to resolve the leak observed.

The present study has some limitations. It is retrospective in nature, the number of participants is small, and the follow-up period is short limiting the statistical power of the analysis. Nonetheless, it represents a comparative case-control study with good data quality and significantly similar groups.

In conclusion, the present study suggests that the safety profile of trabeculectomy with Healaflow has not been shown to be inferior to the one of the trabeculectomy alone. In addition, we did not observe any advantages over traditional trabeculectomy in terms of bleb encapsulation incidence and hypotensive effect during our 6-month follow-up period. As with the occurrence of early hypotony (IOP < 6 mmHg), the frequency was similar in both groups (none), and we consider that it has to do more with the consistency of the scleral flap suturing by the surgeon and less with the space-occupying effect of the agent.

Taking into account the short-term results of our study and the relatively high price of this new agent we should be cautious when considering its usefulness in glaucoma incisional surgery. Of course, prospective studies with larger number of patients and longer follow-up are required to confirm the above findings and also to examine the long-term outcomes of trabeculectomy with Healaflow.

## Figures and Tables

**Table 1:** The technical sheet of Healaflow.

Concentration	22.5 mg/mL
Molecular weight	>2.5 Mda
Cross-linking agent	1,4-Butanediol diglycidyl ether
Origin of the polymer	Nonanimal/biofermentation
pH	Physiological: 7.0
Osmolarity	Physiological: 305 mOsm/kg
Endotoxin content	0.5 EU/mL
Low protein rate	<50 ppm
Sterilization	Autoclave sterilization

**Table 2:** Demographic characteristics of the study group (patients who underwent trabeculectomy with Healaflow) and the control group (patients who underwent trabeculectomy).

	Study group	Control group	*P* value
Number of eyes	20	20	
Age (yrs)			
Mean (±SD)	62.9 (±15.8)	64.15 (±13.3)	0.796
Range	22–81	29–80	
Median	70.5	67	
Gender			
Male	7 (35%)	9 (45%)	
Female	13 (65%)	11 (55%)	0.518
Eye laterality			
Right	8 (40%)	11 (55%)	
Left	12 (60%)	9 (45%)	0.17
Diagnosis			
POAG	10 (50%)	9 (45%)	
PXG	5 (25%)	6 (30%)	
PG	1 (5%)	1 (5%)	
Pseudophakic G	2 (10%)	2 (10%)	
PACG	2 (10%)	2 (10%)	0.915
Preoperative IOP (mmHg)			
Mean (±SD)	29.2 (±5.61)	28.7 (±8.85)	0.815
Range	20–42	19–50	
Number of preoperative medications			
Mean (±SD)	3.4 (±0.75)	3.6 (±0.6)	0.358
Range	2–4	2–4	

**Table 3:** Postoperative complications for the study (patients who underwent trabeculectomy with Healaflow) and the control (patients who underwent trabeculectomy) groups.

	Study group	Control group	*P* value (2-tailed)	*P* value (1-tailed)
Hypotony	1 (5%)	1 (5%)	1	0.744
Flat anterior chamber	1 (5%)	2 (10%)	1	0.5
Hyphemas	1 (5%)	2 (10%)	1	0.5
Positive Seidel test	1 (5%)	0 (0%)	1	0.5
Encapsulated bleb	4 (20%)	5 (25%)	1	0.5

**Table 4:** Number of 5-FU injections performed in the study and control groups and change in intraocular pressure (IOP) before and after treatment.

	Number of patients	Initiation of therapy	Number of injections	Total dose (mg)	IOP before treatment (mmHg)	IOP after treatment (mmHg)
Study group	4 (20%)	7th PD	2	10	19	15
		15th PD	4	20	20	14
		15th PD	1	5	21	16
		1st PM	7	35	28	18

Control group	5 (25%)	7th PD	2	10	24	18
		15th PD	2	10	19	12
		15th PD	8	40	30	17
		15th PD	6	30	24	18
		1st PM	5	25	35	18
